# 

**Published:** 1860-10

**Authors:** 


*Delay of the Present Number.*—We must ask the indulgence of our readers for the delay of the present number. The melancholy cause is already known to them. For all the purely dental articles, and for much of the other matter, they are indebted to the kindness of Dr. A. A. Blandy, who has given no little time and attention to it.

### INDEX.—VOL. XI.

PAGE.

A.

Artificial Dentures, . . . 16
Anchylosis of the Jaws, &c. 226
Antimony in Gold, . . . 298
Arctic Expedition, . . . 302
Anæsthesia and Anæsthetics, 363
Appeal of the Widow of Horace Wells, . . . . . 389
Asphyxia from Chloroform . 435
Arsenic Poisoning and Arsenic Eating, . . . . . 448
Apology, . . . . 454
Allen, Dr. J., Artificial Dentures, 16
Arsenic Eating, . . . . 513
Acupressure for the Arrest of Hemorrhage, . . . 561
Anæsthetic Electricity as an, . 594
Axillæ, Milk Secreted by, . 569
Arsenical Mineral Waters, . 581
Anatomy of Fifth Pair of Nerves, 586
Adulterations, . . . 592
Appointments, Professional, . 593

B.

Barbour, J. H., M. D., Diptherite, 382
Bernard, Claud. Study of the Parotid Gland . . . 414
Bread, Aerated, . . . 428
Burnham, H. B., M. D. Salivary Calculus, . . . 437
Base, Vulcanite, . . . 470
Bad Breath, . . . . 596
Brodie, Sir Benjamin, . . 596
Baillie, Dr. . . . . 596
Baltimore College, Change in Faculty, . . . . . 598

C.

Case, . . . . . 26
Colburn, G. F. J. Reproduction, &c., . . . . . 62
Chloroform, . . . . 69

PAGE.

Cancer, Extirpation of the entire Lower Lip, . . . 133
Case of Adipocere, . . 142
Crania, . . . . 147
Contributors, . . . . 148
Casting Metallic Dies Direct, &c., 183
Clark, F. Y., D. D. S. Making Dies, . . . . 234
Corrections, . . . . 242
Cavities in Filling Teeth, . 325
Cartwright's Inaugural Address, 334
Chloroform Faradization, &c., 435
Currents and Counter Currents in Medical Science, . . . 447
Creasote, . . . . 481
Cavities, Filling of, . . . 510
Chloroform, on the Nature of Death from, . . . 551
Chloroform. Death from, . 555
Chloroform Liniment, . . 595
Ceratitis, . . . . 595
Cancer, Death from Inoculation, 598
Courts, Power of, over Medical Witnesses, . . . . 598
Cholera, . . . . 598
Convention, Medical, . . 594

D.

Dental Anomalies, &c., . . 3
Dentures, Artificial, . . . 16
Decay, Superficial, &c., . . 28
Decay, Dental, . . . . 71
Dentistry, Practical, . . . 176
Dental Profession. Hints to, &c., 181
Dies, Improvement in Casting, 183
Dies, A New Method, &c., . 234
Debray, H., Platina and Metals, 193
Distressing Nervous Symptoms, &c., . . . . . 307
Differences in Gold Foil, . 322
Dental Surgery, School of, . 334
Diptherite, . . . . 382
Dietitic Value of Fermented and Aerated Bread, . . . 428

PAGE.

Danglish, J., M. D., . . 428
Dental Education . . 446
Dentine, Treatment of Sensitive 467
Dentistry, the Practical Utility of, 475
Dentistry, Artificial, and Patents, 489
Death, on the Nature of, from Chloroform, . . . 551
Dental Society, . . 596
Dentists and R. C. of S., . 598
Death from Inoculation of Virus, 598
Delay of Number, . . 598
Delirium Tremens, Treatment of, 583
Diploma, Revoking of, . . 597
Digitalis in Delirium Tremens, 583
Dislocation, Painless Reduction of, 592

E.

Erratum, . . . 148

Exsection of Superior Maxilla, 371

Evils of Tight Lacing, . . 412

Education, Dental, . . 446

Eating, Arsenic, . . 448

Education, Physical, . 487

Electricity as an Anæsthetic, 594

F.

Filling Teeth, . . . 28

Formation of Shells, Bones, &c., 59

Fumes of Phosphorus, &c., . 64

Forget, A. M., M. D. Dental Anomalies, . . 149

Forming Cavities, . . 325

Face, Reparation of, &c., . 330

Fœtid Perspiration, . . 404

Faradization in Asphyxia, . 435

Facial Neuralgia, . . 498

Fractures of Inferior Maxilla, 504

Filling Carious Cavities, . 510

Fang Filling, . . 597

Filling Fangs, . . . 597

Faculty Change of, . . 598

Fractures of Superior Maxilla, 577

Fifth Pair of Nerves, . 586

G.

Gorgas, Dr. F. Case, . . 26

Growth of the Incisors in Rodents, 30

Glands, Salivary, . . 212

Gold Containing Antimony, 298

Gold Foils, the Difference, &c. 322

Gland, Extirpation of Parotid, 539

Gangrene Treated by Kreasote, 584

Gutta Percha as a Permanent Stopping, . . . . 586

H.

Harvey, Dr., . . . 57
Hayward, G., M. D., . 124

PAGE.

Hints and Suggestions to the Dental Profession, . . 181
House, Dr. J. Carroll. Improved Dies, . . 183
Hypnotism, . . . 292
Harvey, Leon F., M. D., . 312
Hints, Practical, . . 316
Hoopes, Wm. H., D. D. S., Reparation of, &c., . . 330
Hulme's Mr., Lectures, . 353
Hamilton Prof. F. H., Paralysis, 406
Hypochlorite of Lime, . 438
Hospital, Long Island, . . 406
Harriss, Dr. W. A., . 504
Hemorrhage, for the Arrest of, 561
Harris, Prof. C. A., Obituary of, 588
Homeopathic College, . 593
Hypnotism, . . . . 592

I.

Influence on the Production of Disease, . . . . 3
Incisors in Rodents, . . 30
Inaugural Address, . . 82
Injection Submucous, for the Cure of Tooth-ache, . . 138
Improved Method of Casting Dies, 183
Inaugural Address, . . 334
Interstitial Keratitis, . . 419
Impressions, Plaster, . 597
Indian Medical Journal, . 597

J.

Jaw Lower, Necrosis of, from, &c. 64
Jaws, Tumors of, &c., . 105
Jaws, Anchylosis of, &c., . 226
Jigger, the . . . 423
Journale de Chimie Medicale, . 454

K.

Keratitis, Interstitial, . . 419
Kreasote in Gangrene, . 584

L.

Letter from Dr. Harvey, . 57
Lip, Lower, Extirpation of, &c., 133
Longevity, . . . . 146
Lower Jaw, Anchylosis of, &c., 226
Levison, Dr. J., . . 307
Loosening of Plates, . 314
London School of, &c., . . 334
Lectures, Mr. Hulmes . 353
Liniment of Chloroform, 595

M.

McCalla, J., D. D. S., . 28

PAGE.
Metropolitan School of D. S., . 82
Maxilla, Resection of, &c., 133
Metals which Accompany Platina, 193
Maryland and Virginia Medical and Surgical Journal, . 303
Maxillary, Exsection of, &c., . 371
Movement Cure, . . 472
McElroy, Dr. C., Neuralgia, 498
Maxilla Fractures of Inferior, 504
Manna, of the Hebrews, . 562
Mallet, the . . . . 597
Milk, Secreted by Tumors in the Axillæ, . . . 569
Maxilla, Fractures of Superior, &c. 577
Medical Convention, . . 594

N.

Necrosis of the Lower Jaw, &c. 64

Necrosis-Phosphor, . . 67

New Method for Dies, . 234

Newspaper Literature, . . 299

Necrology, . . . . 303

Nervous Symptoms in Smokers, 307

Naples and Salerno, . . 387

New Medical Journal, . 454

Neuralgia Facial, &c., . . 498

Needle for Sutures, . . 594

New Disease of the Gums, . 592

O.

On Filling Teeth, . . 28
On Continued Growth of the Incisors, &c., . . . . 30
On the Formation of Shells and Bone, . . . 59
On Dental Decay, . . 71
Our Contributors, . . 148
On Tic Douloureux, . . 254
On the Action of Hypochlorite, 438
Oudet, J. E., . . . 30
Organic Matter, Influence of Sun's Rays upon, . . . 557
Orum & Armstrong's Teeth, 593

P.

Phosphor Necrosis, . . 67
Phosphorous, Fumes of, &c., . 64
Pregnancy, Tooth-ache in, &c. 138
Practical Dentistry, . . 176
Plaster Impressions, . . 183
Profession in England, . 250
Platina and its Metals, 301, 514
Plates, Loosening of, . . 314
Practical Hints, . . 316
Palate, Reparation of, . . 330
Professional Pecksniffs, . 410
Parotid Gland, . . . 414
Proceedings of the Massachusetts Medical Society, . . 439

PAGE.
Physical Education, . . 487
Patents and Artificial Dentistry, 489
Parotid Gland, Extirpation of, 539
Psychology, . . . 595
Plaster Impressions, . . 597
Platinum, . . . 596
Pivot Teeth, . . . 596

Q.

Quackery, and who are, &c., 20

R.

Reproduction of Canine Teeth, 62
Richardson, Dr., Address, . 82
Report on Tumors of the Jaws, 105
Remarks on Anæsthesia, . 124
Resection of, &c., . . 133
Remarkable Case of Adipocere, 142
Reparation of the Face, and Palate, 330
Revoking a Diploma, . 597
R. C. of S. Dentists, and the, 598

S.

Societies, Dental, . . 42
Shells, Formation of, &c. . 59
Submucous Injection, . . 138
Salivary Glands, . . 212
Surgery, Tomes, . . 148
Shepherd, S. M., on Patents, 489
Sutures, Novel Needle for, . 594
Scrofula, . . . . 595
Surgery, Principles and Practice of, . . . . 586

T.

Thomson, F. H., M. D., . 71
Tumors, Report on, &c., . 105
Tooth-ache of Pregnancy, . 138
Tomes' Dental Surgery, . 148
Taft's Operative Dentistry, . 148
Thompson, T. D., D. D. S., 181
Tobacco—its Effects upon the Health and Teeth, . . 455
Treatment of Sensitive Dentine, 467
Trephine, a new, . . 596
Teeth, Pivot, . . 596
The Mallet, . . . 597
Teeth. Orum & Armstrong's, 593

V.

Veratrine—its Use in Dental Surgery, . . . . 312
Vulcanite Base, . . . 470

W.

Witnesses, Power of Courts over, 598

